# Hospitalization Rates and Comorbidities in Patients with Progressive Supranuclear Palsy in Germany from 2010 to 2017

**DOI:** 10.3390/jcm9082454

**Published:** 2020-07-31

**Authors:** Maria Angela Samis Zella, Dirk Bartig, Lennard Herrmann, Gesine Respondek, Günter Höglinger, Ralf Gold, Dirk Woitalla, Christos Krogias, Lars Tönges

**Affiliations:** 1 Department of Neurology, St. Josef-Hospital, Ruhr-University Bochum, 44801 Bochum, Germany; s.zella@contilia.de (M.A.S.Z.); lennard.herrmann@rub.de (L.H.); ralf.gold@rub.de (R.G.); dirk.woitalla@rub.de (D.W.); christos.krogias@rub.de (C.K.); 2 Department of Neurology, St. Josef-Hospital, Katholische Kliniken Ruhrhalbinsel, Contilia Gruppe, 45257 Essen, Germany; 3 DRG MARKET, D-49069 Osnabrück, Germany; dirk.bartig@drg-market.de; 4 Department of Neurology, Hannover Medical School, 30625 Hannover, Germany; Respondek.Gesine@mh-hannover.de (G.R.); Guenter.Hoeglinger@dzne.de (G.H.); 5 German Center for Neurodegenerative Diseases (DZNE), 81377 Munich, Germany; 6 Department of Neurology, Technical University of Munich, 81675 Munich, Germany; 7 Neurodegeneration Research, Centre for Protein Diagnostics (ProDi), Ruhr University, 44801 Bochum, Germany

**Keywords:** progressive supranuclear palsy, PSP, Parkinson’s disease, Parkinsonian syndromes

## Abstract

Progressive supranuclear palsy (PSP) belongs to the disease spectrum of Parkinsonian syndromes. Due to the chronic and progressive neurodegenerative course of the disease, PSP patients often have to be hospitalized to undergo diagnostic and therapeutic measures. The dynamics and characteristics of PSP inpatient treatment in Germany have not been investigated thus far. The current study analyzed trends of inpatient treatment in Germany for the years 2010–2017 based on the German DRG statistics (“diagnostic-related groups”) in the category G23.- (other degenerative diseases of the basal ganglia) and with special focus on PSP (G23.1). Inpatient case numbers of the G23.- category comprised a total of 21,196 patients from 2010–2017, whereas the PSP subcategory (G23.1) amounted to 10,663 cases. In the analyzed time period, PSP patient numbers constantly increased from 963 in 2010 to 1780 in 2017 with yearly growth rates of up to 20%. Similar trends were observed for other Parkinsonian syndromes such as multiple system atrophy (MSA). Differentiating PSP inpatients by gender demonstrated a higher proportion of males (55–60%) in comparison to female patients for the entire observation period. The average age of hospitalized PSP patients over these years was between 72.3 and 73.4 years without relevant differences for gender. The most common comorbidities consisted of cardiovascular, neurological, muscular and urological disorders. In summary, the analysis demonstrates that PSP patients are increasingly hospitalized in Germany and the current concepts of stationary care have to differentiate standard practices for Parkinson’s disease (PD) to also address the needs of patients with PSP and other Parkinsonian syndromes.

## 1. Introduction

Progressive supranuclear palsy (PSP) and Parkinson´s disease (PD) both belong to the disease spectrum of Parkinsonian syndromes (PS). PD is primarily characterized by bradykinesia, muscular rigidity, tremor and gait impairment with postural instability. The predominant clinical phenotypes are a tremor-dominant subtype, a postural instability and gait disorder dominant subtype [1]. In addition, there exist classifications for phenotypes with different progression rates of disease (mild motor-predominant, intermediate, diffuse malignant) [2]. Concerning PSP, several clinical subtypes have been defined in the recently-updated Movement Disorder Society-endorsed clinical diagnostic criteria, such as PSP with parkinsonism (PSP-P), PSP with progressive gait freezing (PSP-PGF) and PSP with Richardson´s syndrome (PSP-RS) [3]. Four functional domains (ocular motor dysfunction, postural instability, akinesia and cognitive dysfunction) have been identified as clinical predictors of PSP and three additional clinical features (within each of these domains) contribute to different levels of diagnostic certainty (probable PSP, possible PSP and suggestive of PSP) [3,4]. The goal of the improved diagnostic criteria is to optimize an early and sensitive diagnosis of PSP but also to enable a more specific clinical diagnosis of PSP on the basis of currently available evidence. This shall help to optimally implement novel disease-modifying treatment approaches that are currently being studied in clinical trials [5,6].

While there have been several publications describing the rising prevalence of PD in Germany [7,8,9,10] and worldwide [11,12], there is no public data available for the prevalence of PSP in Germany. Several small studies have been performed in other countries mostly at a county level, which preferentially considers the more common PSP-RS variant. In Switzerland, a descriptive cross-sectional prevalence study for Parkinsonism including PD and PSP has been conducted in the Canton of Geneva showing a crude prevalence of PSP between 5.9 and 11.3 per 100,000 inhabitants [13]. The study of Golbe et al. in two New Jersey counties in the US yielded a prevalence of 1.39 [14]. Two Japanese studies collected epidemiological PSP data of all phenotypes in Yonago City where the prevalence of 5.8/100,000 in 1999 [15] rose to 17.9/100,000 in 2010 [16]. In London, UK, 6.4 PSP cases per 100,000 were detected based on records of general practices [17] and in a more recent analysis in two UK counties for frontotemporal lobar degeneration syndromes, PSP was found to have a prevalence of up to 18 per 100,000 people in the age group between 70 and 74 [18]. It is expected that the application of the new clinical diagnostic criteria for PSP will substantially increase the number of overall diagnoses by the inclusion of more non-PSP-RS forms of PSP in the future [19].

The current study aims to evaluate the number of inpatient treatment for PSP patients in Germany. For the first time, an in-depth analysis of the characteristics and dynamics of PSP inpatient treatment in Germany is provided for the years 2010–2017 based on the German DRG statistics (“diagnostic-related groups”). The characterization includes the differentiation of age and gender groups as well as the presence of comorbidities. As the inpatient treatment of PSP patients is continuously increasing, this might imply a need for strengthening of the currently available resources for diagnosis and treatment of PSP in Germany.

## 2. Methods

Analyses were based upon the evaluation of the German Diagnosis-Related Groups (G-DRG) data from 2010 to 2017, provided by the German federal statistical office for all districts of Germany (DRG-statistic, www.destatis.de). All inpatients admitted to a German hospital have to be encoded by the International Statistical Classification of Diseases and Related Health Problems 10th revision, German modification (ICD-10-GM). The codes contain the treatment reason for inpatient admission (main diagnosis) and the documentation of the patient’s comorbidities (secondary diagnoses). The correct use of the codes is based on mandatory regulations, such as the German Coding Guidelines and recommendations of the Medical Service of the Health Insurance Funds, and is closely supervised.

With regard to the study design, in the present analysis, we included all inpatient cases with the main diagnosis of the ICD G23 category (“Other degenerative diseases of the basal ganglia”). We analyzed the following variables: hospitalization trends from 2010 to 2017 of patients with diagnosis G23.-, with special focus on G23.1, age, sex and comorbidities. As the analysis is based on anonymized data, separate case settings were evaluated that can also include repeated admissions of an individual patient if the admissions are so far apart in time that cases are not merged. Detailed subanalyses were performed for the ICD G23.1 subcategory (“Steele-Richardson-Olzewski syndrome”), which is commonly used for the classification of PSP patients in Germany (see Table 1). Current coding of PSP (G23.1), according to the German version of ICD-10, formally refers to Richardson’s syndrome only, but it has to be assumed that in common practice all PSP subtypes are coded under this category. We, therefore, refer to G23.1 as PSP in this manuscript. For each reporting year, we determined the mean age and gender ratio. In addition, the morbidity burden of patients with main diagnosis G23.1 was evaluated on the basis of associated secondary diagnoses.

### Statistical Analyses

Statistical analyses and graphics were performed using Excel 2016 (Microsoft Corporation, Redmond, USA) and R (v 3.3.0-R: A language and environment for statistical computing. R Foundation for Statistical Computing, Vienna, Austria. URL http://www.R-project.org/.). As the grouping was done according to gender and G23.- diagnosis, all data were analyzed in relation to gender and to G23.- diagnosis. All computations are shown in Table S1.

## 3. Results

Patient cases with the main diagnosis “other degenerative disease of the basal ganglia” (G23.-) and, in particular, cases with the diagnosis of PSP (G23.1) have been analyzed in detail in the present study. The dynamics of G23.- inpatient treatment from 2010 to 2017 in Germany is presented first followed by differentiation of age and gender groups. Comorbidities were compared between the overall G23.- population and the G23.1 group.

### 3.1. Development of Inpatient Case Numbers with PSP (G23.1) between 2010 and 2017

**Figure 1:**
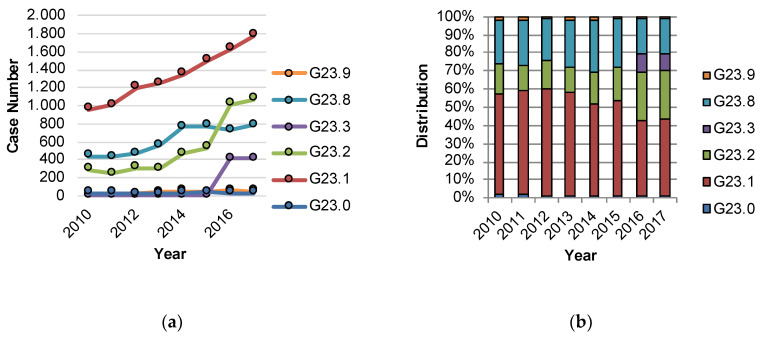
(**a**) Development of inpatient treatment numbers in Germany for patient cases with diagnosis G23.- from 2010 to 2017; (**b**) Relative distribution of subcategories in the main diagnosis G23.- between 2010 and 2017. G23.0: Neurodegeneration with Brain Iron Accumulation, G23.1: Steele-Richardson-Olzewski syndrome (PSP—progressive supranuclear palsy), G23.2: Multiple system atrophy of parkinsonian type (MSA-P), G23.3: Multiple system atrophy of cerebellar type (MSA-C), G23.8: Other specified degenerative diseases of the basal ganglia, G23.9: Other degenerative diseases of the basal ganglia, not further defined.

In 2010, a total number of 1749 patients with Other degenerative disease of the basal ganglia (G23.-) were treated as inpatients in Germany. Of these, the largest proportion, i.e., 55.1% (963 patients), was diagnosed with PSP (G23.1). Treatment numbers for the entire G23.- category increased up to 4120 cases through the year 2017, including 1780 cases with PSP. In 2017, the three most common diagnoses were PSP (G23.1), accounting for 43.2% of the cases (1780 patients), followed by multiple system atrophy of parkinsonian type (MSA-P) (G23.2), which reached a proportion of 26.0% of all the cases (1071 patients), and other specified degenerative diseases of the basal ganglia (G23.8), which were documented as having a proportion of 19.0% (786 patients) (Figure 1). Detailed inpatient numbers of the subgroups G23.0 to G23.9 for the year 2017 are shown in Table 2.

### 3.2. Relative Annual Changes in Inpatient Treatment for G23.- Disease Subgroups 

**Figure 2:**
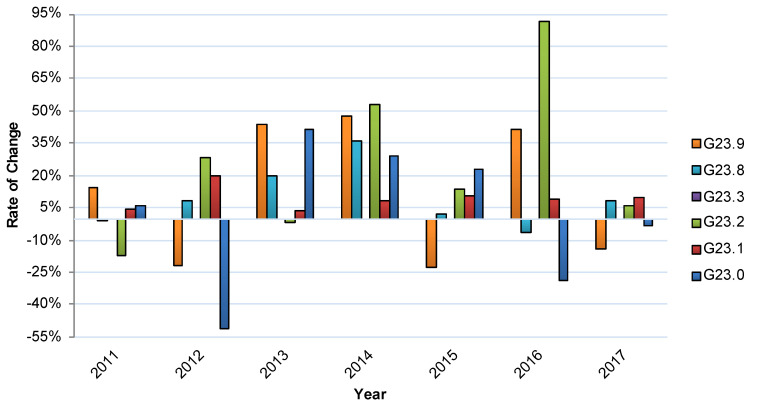
Relative annual changes in inpatient treatment for disease subgroups between 2010 and 2017. G23.0: Neurodegeneration with Brain Iron Accumulation, G23.1: Steele—Richardson—Olzewski syndrome (PSP), G23.2: Multiple system atrophy of parkinsonian type (MSA-P), G23.3: Multiple system atrophy of cerebellar type (MSA-C), G23.8: Other specified degenerative diseases of the basal ganglia, G23.9: Other degenerative diseases of the basal ganglia, not further defined.

The dynamics in inpatient case numbers for the G23.- subcategories was very heterogeneous over the years 2010 to 2017. While for the diagnosis Neurodegeneration with Brain Iron Accumulation (G23.0) there were very small patient numbers overall, the largest proportion of PSP (G23.1) showed continuous increases, with up to 19% per year. Multiple system atrophy of parkinsonian type (MSA-P) (G23.2) rises strongly from 2015 to 2016. The subcategory multiple system atrophy of cerebellar type (MSA-C) (G23.3) was additionally introduced into the German ICD-10 version only in 2016. Case numbers for other specified degenerative diseases of the basal ganglia (G23.8) remained rather stable except for a strong decrease in 2012 and a subsequent rise in 2013 (Figure 2).

### 3.3. Characterization of PSP (G23.1) Inpatients

**Figure 3:**
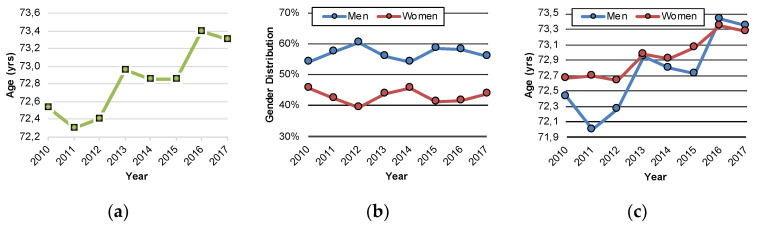
Dynamics of PSP (G23.1) inpatient case numbers and gender distribution from the year 2010 to 2017 in Germany. (**a**) Distribution of diagnoses according to gender; (**b**) distribution of diagnoses according to age at examination; (**c**) mean age of patients at examination differentiated by gender.

The inpatient case number for PSP (G23.1) amounted to 963 in 2010 and 1780 in 2017, indicating a growth rate of 85% over seven years. The number of inpatients with a diagnosis of PSP (G23.1) constantly increased each year, with the strongest increase between 2011 and 2012 (Figure 3a). If differentiated by gender, the PSP (G23.1) inpatient analysis demonstrated a greater proportion of male patients over the entire observation period. The highest male proportion was found for 2012, with 60.6%, and in 2015, with 58.1 % (Figure 3b). Concerning the mean age of PSP (G23.1) inpatients at the examination, there were only minor alterations between mean ages of 72.3 and 73.4 years, for the years 2010 to 2017 (Figure 3c).

### 3.4. Comorbidities

**Figure 4:**
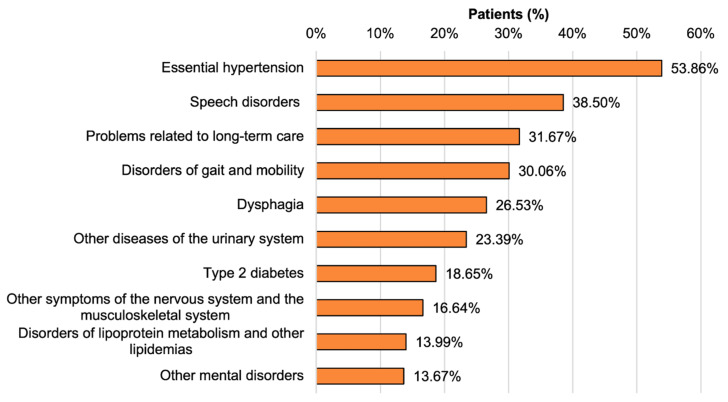
Prevalence of the 10 most frequent comorbidities among patients with PSP (G23.1) in 2017.

The main comorbidities of PSP (G23.1) inpatients consisted of cardiovascular, neurological, muscular and urological disorders. The 10 most frequently observed diagnoses were essential hypertension, speech disorders, problems related to long-term care, gait and motility disorders, dysphagia, diseases of the urinary system, diabetes mellitus type II, symptoms of the nervous and musculoskeletal system, disorders of lipoprotein metabolism and other mental disorders (Figure 4).

Importantly, essential hypertension was diagnosed in 54% of the patients. Comorbidities of neuro-psychiatrical disorders (speech disorders, gait and motility disorders, dysphagia, symptoms of the nervous and musculoskeletal system and other mental disorders) were present in a range from 14% to 39% for all patients. Metabolic disorders such as type 2 diabetes mellitus and disorders of lipoprotein metabolism were found in 19% and 14% of patients, respectively. In comparison to the ratio of comorbidities in patients within the German DRG diagnostic category Primary Parkinson’s syndrome (G20.-) in 2017, PSP patients (G23.1) showed speech disorders (38.50 % vs. 22.92%) and dysphagia (26.53% vs. 11.66%) substantially more often, but motor dysfunction less often (12.94% vs. 23.75%) (Table S2). The most frequently coded main diagnoses with G23.1 or G23.- as secondary diagnosis in 2017 were pneumonia caused by solid and liquid substances, primary Parkinson´s disease, glaucoma, pneumonia with not specified pathogen, hypovolemia, intracranial injuries, other diseases of the urinary system, epilepsy, stroke and sepsis (Tables S3 and S4). 

**Figure 5:**
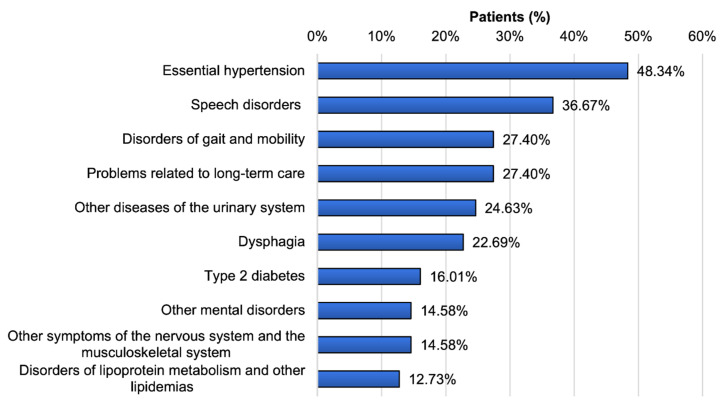
Prevalence of the 10 most frequent comorbidities among patients in the diagnostic group G23.- in 2017.

The distribution of comorbidities for patients with a diagnosis in the G23.- principal category was similar to the comorbidity distribution for patients with diagnosis G23.1, including cardiovascular, neurological, muscular and urological disorders. Essential hypertension was diagnosed in 48% of these patients. Comorbidities in the form of neuro-psychiatrical disorders (speech disorders, gait and motility disorders, dysphagia, other mental disorders due to damage or dysfunction of the brain or physical illness and symptoms of the nervous and musculoskeletal system) were present in a range from 13% to 37% of the patients (Figure 5).

## 4. Discussion

The current analysis evaluates the development of hospital treatment for patients in Germany with the diagnostic category G23.- (“Other degenerative diseases of the basal ganglia”), based on data from the DRG statistics (“diagnostic-related groups”). We here provide for the first time an in-depth analysis of the characteristics and dynamics of PSP (G23.1) inpatients in Germany for the years 2010–2017.

The inpatient case numbers with main diagnosis G23.- in Germany doubled from 2010 to 2017, from 1749 to 4120 inpatients, demonstrating a substantial increase of 136%. This is in line with a strong rise of inpatient hospitalization with a diagnosis of PD in Germany. Especially patients with motor fluctuations (G20.01 and G20.11) had to be increasingly hospitalized between 2010 and 2015 with case number progressions of more than 50% [9]. In our current analysis, the PSP subgroup (G23.1) represents the most numerous proportion of the G23.- category with 1780 patient cases in 2017. Other subpopulations from the G23.- group also presented strong increases of inpatient treatment, with 1071 patient cases for multiple system atrophy of parkinsonian type (MSA-P; G23.2) and 786 patient cases of other specified degenerative disease of the basal ganglia (G23.8).

Explanations for why an increased number of inpatient PSP treatments were necessary between 2010 and 2017 could be due to the fact that PSP is diagnosed more frequently because of increasing knowledge of the disease. Both neurologists and movement disorder specialists could be more aware of the diagnosis and identify more patients. In addition, patients and their advocacy groups spread their knowledge to the community that may motivate people under suspicion of diagnosis to consult specialist neurologists. The availability of studies with novel treatment approaches and the very dynamic patient cohort programs in Germany (ProPSP, DescribePSP) further facilitate patient access. Fortunately, once identified, patients will be able to receive inpatient treatment at specialized centers to deal with disease exacerbations or worsened comorbidities.

The analysis of gender distribution resulted in a greater proportion of the PSP diagnosis for male patients over the entire observation period with a maximum peak in 2012 with 60.6% male patients. In the analysis of Takigawa et al., comparable rates for gender distribution with more male patients affected were observed [16]. In other studies, these data were not sufficiently documented [16,20,21]. The mean age of hospitalized PSP patients only varied between 72.3 and 73.4 years over the entire period of analysis. In the aforementioned cross-sectional Japanese study, patient ages were in the range of 77.5 ± 6.5 years [16].

In our PSP patient population of the year 2017, the most frequently observed comorbidities were essential hypertension, speech disorders, problems related to long-term care, gait and motility disorders, dysphagia, diseases of the urinary system, diabetes mellitus type 2, symptoms of the nervous and musculoskeletal system, disorders of lipoprotein metabolism and other mental disorders. Essential hypertension was observed in 54% of the patients with the main diagnosis G23.1. Metabolic disorders such as diabetes mellitus type 2 and disorders of lipoprotein metabolism were documented in a rate of 19% and 14%, respectively. In comparison to age-matched population subgroups, the prevalence of diabetes mellitus and metabolic syndrome among patients with diagnosis G23.-/G23.1 is at a similar level, whereas G23.-/G23.1 patients have a higher prevalence of essential hypertension (54% vs. 5–34% in the age-matched healthy population) [22,23]. The high rate of vascular and metabolic disorders (essential hypertension, type 2 diabetes mellitus and disorders of lipoprotein metabolism) suggests that PSP patients should routinely be evaluated for these comorbidities in order to avoid the contribution of other factors that may increase the neurodegenerative burden to the CNS and could facilitate gait disorders through the development of, e.g., peripheral artery disease or polyneuropathy. Importantly, PSP patients show a different spectrum of motor difficulties than, e.g., Parkinson´s patients, which has to be considered in the planning of the individual treatment focus.

In order to prioritize the most important pharmacological and also non-pharmacological treatment approaches for patients, a precise clinical evaluation is necessary that can be performed with the recently developed Progressive Supranuclear Palsy Clinical Deficits Scale [24]. The detection of individual deficits in seven clinical domains can assist in the selection of accurate inpatient treatment concepts, such as multimodal complex treatment that is offered in many hospitals throughout Germany [25].

A limitation of this study is that the data are based on documented diagnoses of the German DRG system and the diagnostic accuracy relies on the coding physicians of the treating hospital. The correctness of diagnoses is regularly monitored by insurance companies based on the written documentation of hospital treatment. The analysis is based on anonymized case data and does not allow to analyze hospitalizations, which may have occurred repetitively for the same patient. However, the creation of a new case for the same patient is only possible if a predefined period between two hospitalizations has clearly been exceeded or a completely new treatment indication has been made. The data do not contain information about the admission reason of the patient. However, this can be derived from the secondary diagnoses or comorbidities that are included in our analysis. Overall, up to 20% of all coded cases are controlled and are degraded in payment if inadequately processed. Therefore, we assume a rather high degree of accuracy. Furthermore, we employ the data of seven subsequent years, providing a robust database for our analyses.

## 5. Conclusions

The impact of Parkinsonian disorders on the health care system is constantly increasing and this includes typical Parkinson´s syndromes, but also atypical forms such as PSP. In this study, we demonstrate a substantial rise in inpatient PSP treatment in Germany and recognize the need for more specialized care for these patients. Neurological clinics need to be better prepared for the treatment not only of PD patients but also of other Parkinsonian syndromes, such as the atypical forms, since the needs of patients can be very different. We expect a further rise in the prevalence of these syndromes, as improved and more sensitive clinical as well as diagnostic tools have been developed that facilitate the diagnosis. This is all the more important as causal treatment options are currently under development that should be offered to patients with such serious diseases.

## Figures and Tables

**Table 1:** Overview of G23.- category and corresponding codes according to the national German DRG (German Diagnosis-Related Groups) classification (ICD-10-GM).

ICD-10	Diagnosis
G23.-	Other degenerative diseases of the basal ganglia
G23.0	Neurodegeneration with Brain Iron Accumulation
G23.1	Steele–Richardson–Olzewski syndrome (PSP)
G23.2	Multiple system atrophy of parkinsonian type (MSA-P)
G23.3	Multiple system atrophy of cerebellar type (MSA-C)
G23.8	Other specified degenerative diseases of the basal ganglia
G23.9	Other degenerative diseases of the basal ganglia, not further defined

**Table 2:** Prevalence of various inpatient subcategories of main category G23.- in 2017.

ICD-10	Diagnosis	Cases, *n*	Distribution, %
G23.-	Other degenerative diseases of the basal ganglia	4120	100
G23.0	Neurodegeneration with Brain Iron Accumulation	26	0.6
G23.1	Steele–Richardson–Olszewski syndrome (PSP)	1780	43.2
G23.2	Multiple system atrophy of parkinsonian type (MSA-P)	1071	26.0
G23.3	Multiple system atrophy of cerebellar type (MSA-C)	407	9.9
G23.8	Other specified degenerative diseases of the basal ganglia	786	19.1
G23.9	Other degenerative diseases of the basal ganglia, not further defined	50	1.2

## Supplementary Materials

The following are available online at https://www.mdpi.com/2077-0383/9/8/2454/s1. Table S1: Overview of the analyzed statistical variables, Table S2: Prevalence of various comorbidities at admission in relation to the diagnosis of G23.1 and G20.- in 2017, Table S3: The 10 most common main diagnoses with G23.1 as a secondary diagnosis in 2017, Table S4: The 10 most common main diagnoses with G23.- as a secondary diagnosis in 2017.
