# Supplementary material for: Hospitalization Rates and Comorbidities in Patients with Progressive Supranuclear Palsy in Germany from 2010 to 2017
Source: J Clin Med. 2020 Jul 31;9(8):2454. doi: 10.3390/jcm9082454 (PMC7465231; doi:10.3390/jcm9082454)
Supplement: Supplementary file 1 [file jcm-09-02454-s001.pdf]

Table S1. Overview of the analyzed statistical variables. G23.-: Other degenerative diseases of the basal ganglia, G23.1: Steele–Richardson–Olzewski syndrome.

Analyzed Variables	Years
Development of inpatient numbers with main diagnosis G23.-	2010-2017
Inpatients number with main diagnosis G23.-	2017
Distribution of subcategories of main diagnosis G23.-	2010-2017
Annual disease distribution changes of inpatient numbers with main diagnosis G23.-	2010-2017
Distribution of main diagnosis G23.1 according to gender	2010-2017
Distribution of main diagnosis G23.1 according to age	2010-2017
Comorbidities main diagnosis G23.1	2017

Table S2. Prevalence of various comorbidities at admission in relation to the diagnosis G23.1 and G20.- in 2017. G.20.-: Primary Parkinson Syndrome. G23.1: Steele–Richardson–Olzewski syndrome.

Comorbidity (ICD-10)	Main Diagnosis G23.1	Main Diagnosis G20.-	Diff.-Points
Essential hypertension (I10)	53.86%	49.17%	4.69%
Speech disorders (R47)	38.50%	22.92%	15.59%
Problems related to long-term care (Z74)	31.67%	30.65%	1.02%
Disorders of gait and mobility (R26)	30.06%	25.98%	4.08%
Dysphagia (R13)	26.53%	11.66%	14.86%
Other diseases of the urinary system (N39)	23.39%	21.74%	1.65%
Type 2 diabetes (E11)	18.65%	16.31%	2.34%
Other symptoms of the nervous system and the musculoskeletal system (R29)	16.64%	13.28%	3.36%
Disorders of lipoprotein metabolism and other lipidemias (E78)	13.99%	12.85%	1.14%
Other mental disorders resulting from brain damage or dysfunction or physical illness (F06)	13.67%	18.20%	-4.54%
Motor dysfunctions (R32)	12.94%	23.75%	-10.81%
Unspecified dementia (F03)	9.89%	2.89%	7.00%
Dementia in other diseases (F02)	7.23%	19.20%	-11.96%
Other extrapyramidal diseases and movement disorders (G25)	4.26%	11.27%	-7.01%

Table S3. Ten most common main diagnoses with G23.1 as secondary diagnosis in 2017. G23.1: Steele–Richardson–Olzewski syndrome.

Comorbidity (ICD-10)	Main Diagnosis 2017 (cases)	Diagnosis G23.1 2017 (cases)	Rate
Pneumonia caused by solid and liquid substances (J69)	33600	99	0.29%
Primary Parkinson´s disease (G20)	45370	75	0.17%
Glaucoma (H40)	74628	49	0.07%
Pneumonia, pathogen not specified (J18)	237839	45	0.02%
Hypovolemia (E86)	125835	44	0.03%
Intracranial injury (S06)	282838	42	0.01%
Other diseases of the urinary system (N39)	152733	39	0.03%
Femur fracture (S72)	188000	38	0.02%

Epilepsy (G40)	146861	31	0.02%
Stroke (I63)	258927	31	0.01%

Table S4. Ten most common main diagnoses with G23.- as secondary diagnosis in 2017. G23.-: Other degenerative diseases of the basal ganglia.

**Comorbidity (ICD-10)**	**Main Diagnosis 2017 (cases)**	**Diagnosis G23.- 2017 (cases)**	**Rate**
Primary Parkinson´s disease (G20)	45370	190	0,42%
Pneumonia caused by solid and liquid substances (J69)	33600	127	0,38%
Stroke (I63)	258927	98	0,04%
Epilepsy (G40)	146861	81	0,06%
Hypovolemia (E86)	125835	79	0,06%
Pneumonia, pathogen not specified (J18)	237839	79	0,03%
Intracranial injury (S06)	282838	70	0,02%
Femur fracture (S72)	188000	65	0,03%
Other diseases of the urinary system (N39)	152733	63	0,04%
Other sepsis (A41)	122574	51	0,04%
